# Signaling Pathway of Taurine-Induced Upregulation of TXNIP

**DOI:** 10.3390/metabo12070636

**Published:** 2022-07-11

**Authors:** Hideo Satsu, Yusuke Gondo, Hana Shimanaka, Masato Imae, Shigeru Murakami, Kenji Watari, Shunichi Wakabayashi, Sung-Joon Park, Kenta Nakai, Makoto Shimizu

**Affiliations:** 1 Department of Biotechnology, Faculty of Engineering, Maebashi Institute of Technology, Gunma 371-0816, Japan; m1281045watari@gmail.com; 2 Department of Applied Biological Chemistry, Graduate School of Agricultural and Life Sciences, The University of Tokyo, Tokyo 113-8657, Japan; glic-gws@hotmail.co.jp (Y.G.); xxx_.87@icloud.com (H.S.); 3 Research & Development Headquarters Self-Medication, Taisho Pharmaceutical Co., Ltd., Tokyo 170-8633, Japan; m-imae@taisho.co.jp; 4 Department of Bioscience and Biotechnology, Fukui Prefectural University, Fukui 910-1195, Japan; murakami@fpu.ac.jp; 5 Human Genome Center, The Institute of Medical Science, The University of Tokyo, Tokyo 108-8639, Japan; shunichi.wakabayashi@gmail.com (S.W.); sjpark@ims.u-tokyo.ac.jp (S.-J.P.); knakai@ims.u-tokyo.ac.jp (K.N.); 6 Research Center for Agricultural and Life Sciences, Tokyo University of Agriculture, Tokyo 156-8502, Japan; ms205346@nodai.ac.jp

**Keywords:** taurine, TXNIP, Ets-1, ERK

## Abstract

Taurine, a sulfur-containing β-amino acid, is present at high concentrations in mammalian tissues and plays an important role in several essential biological processes. However, the genetic mechanisms involved in these physiological processes associated with taurine remain unclear. In this study, we investigated the regulatory mechanism underlying the taurine-induced transcriptional enhancement of the thioredoxin-interacting protein (TXNIP). The results showed that taurine significantly increased the luciferase activity of the human TXNIP promoter. Further, deletion analysis of the TXNIP promoter showed that taurine induced luciferase activity only in the TXNIP promoter region (+200 to +218). Furthermore, by employing a bioinformatic analysis using the TRANSFAC database, we focused on Tst-1 and Ets-1 as candidates involved in taurine-induced transcription and found that the mutation in the Ets-1 sequence did not enhance transcriptional activity by taurine. Additionally, chromatin immunoprecipitation assays indicated that the binding of Ets-1 to the TXNIP promoter region was enhanced by taurine. Taurine also increased the levels of phosphorylated Ets-1, indicating activation of Ets-1 pathway by taurine. Moreover, an ERK cascade inhibitor significantly suppressed the taurine-induced increase in TXNIP mRNA levels and transcriptional enhancement of TXNIP. These results suggest that taurine enhances TXNIP expression by activating transcription factor Ets-1 via the ERK cascade.

## 1. Introduction

Taurine (2-aminoethanesulfonic acid) is a free β-amino acid abundant in several tissues, such as muscles, the heart, brain, and eyes. Previous research indicates that taurine performs various functions, including as an antioxidant, osmoregulator, and bile acid conjugate, as well as in detoxification [1,2]. Further, taurine is reported to be essential for the development of fetuses and newborns [3,4].

Taurine is obtained directly from diet and is also synthesized endogenously from cysteine, which in turn is formed from methionine. Dietary taurine is absorbed by intestinal epithelial cells via the taurine transporter (TAUT, SLC6A6). We previously reported that TAUT is regulated by various extracellular conditions, such as adaptive regulation and hyperosmolarity, as well as by tumor necrosis factor-α (TNF-α) [5,6,7]. Further, considering that the intestinal TAUT was regulated by inflammatory cytokines [7], we previously investigated the relationship between taurine and inflammation and reported that taurine reduced colitis symptoms in a mouse model of DSS-induced colitis [8]. Furthermore, using DNA microarrays, we comprehensively analyzed the effects of taurine on overall gene expression in intestinal epithelial cells and found that taurine markedly enhanced the mRNA expression and transcriptional activity of the thioredoxin-interacting protein (TXNIP) [9].

TXNIP suppresses its activity by interacting with thioredoxin [10,11], and various physiological functions have been reported for TXNIP [11]. It is reported that TXNIP knockout mice have significantly reduced adaptability to energy deprivation [12,13,14,15,16] because they develop metabolic abnormalities, such as reduced efficiency of fatty acid metabolism [15], development of hyperlipidemia [13,17], and hypoglycemic states [14,16]. Furthermore, TXNIP contributes to glycogenesis in the liver [18], and the relationship between TXNIP mutations and the development of diabetes and hypertension has been suggested [17,19]. TXNIP has also been shown to regulate cardiac hypertrophy [20]. Furthermore, immune enhancement by moderately strong expression of TXNIP is also considered [11]. Physiological functions in the intestinal tract have been reported by Takahashi et al. [21] that TXNIP mRNA expression in the site of inflammation in patients with ulcerative colitis, is decreased, which is thought to contribute to the development of ulcerative colitis. Additionally, a previous study has shown that taurine regulates the functions of human intestinal Caco-2 cells via TXNIP induction [22]. Thus, taurine regulates the mRNA expression of TXNIP; however, the mechanisms underlying its regulation remain unclear.

In the present study, we analyzed the transcription factors and signaling molecules involved in the taurine-induced increase in the transcriptional activity of TXNIP.

## 2. Results

### 2.1. Effect of Taurine on Luciferase Activity Involving the TXNIP Promoter Region

**Figure 1:**
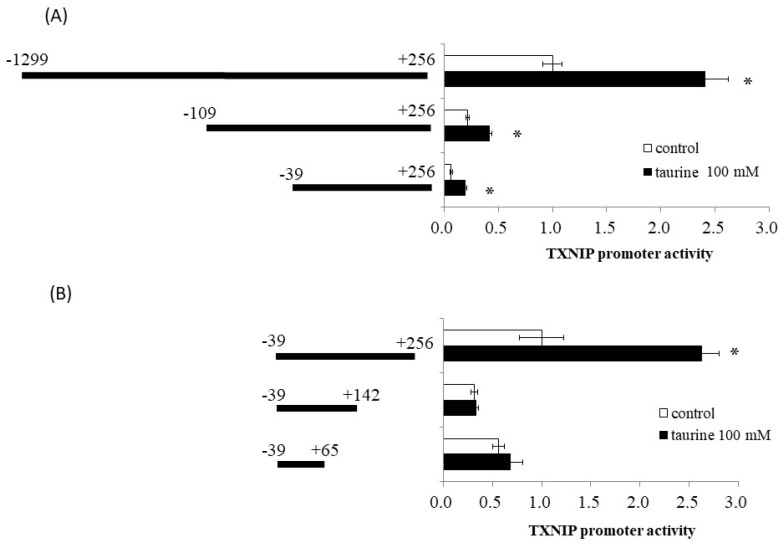
Effect of taurine on the transcriptional activity of the TXNIP promoter between −1299/+256. Caco-2 cells were transfected with a reporter vector containing partial promoter regions of TXNIP and then replaced with a medium containing 100 mM of taurine. After 24 h, the cells were subjected to a luciferase assay, as described in Materials and Methods Section 4.4. (**A**) TXNIP promoter partial sequences (−1299/+256, −109/+256, −39/+256). Results are expressed as relative values with the control value of (−1299/+256) as 1. (**B**) TXNIP promoter partial sequences (−29/+256, −29/+142, −39/+65). Results are expressed as relative values with the control value of −39/+256 as 1. Each value represents the mean ± S.E. (*n* = 3), * *p* < 0.05, vs. the control value (Student’s *t*-test).

Using reporter analysis, we previously reported that taurine increased the promoter activity of TXNIP [9]. A reporter vector containing the promoter region of TXNIP (−1299/+256) was used. Therefore, we examined the effect of taurine on promoter activity in various regions of the TXNIP promoter, including (−1299/+256), (−109/+256), and (−39/+256), with truncation of the 5′-flanking region. As shown in Figure 1A, taurine increased the promoter activity of all three reporter vectors, suggesting that the promoter region (−39/+256) is essential for the taurine-induced increase in TXNIP promoter activity.

We also constructed a reporter vector containing the (−39/+256), (−39/+142), and (−39/+65) sequences of the promoter region of TXNIP. Taurine markedly increased reporter activity in the presence of a reporter vector containing the promoter region between (−39/+256), but did not increase activity in the case of (−39/+142) and (−39/+65) (Figure 1B). This result suggests that a taurine response element is in the TXNIP promoter (+143/+256).

**Figure 2:**
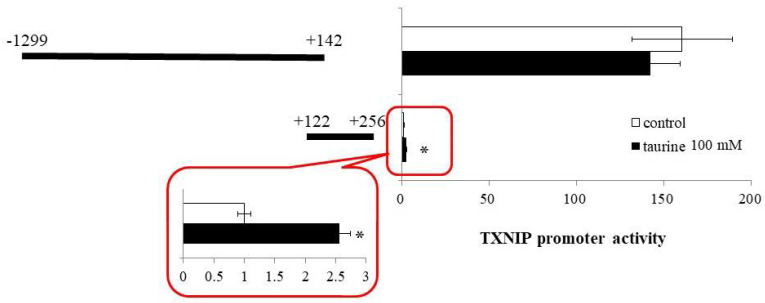
Effect of taurine on the transcriptional activity of the TXNIP promoter containing −1299/+142 and +122/+256. Caco-2 cells were transfected with a reporter vector containing partial promoter region of TXNIP (−1299/+142, +122/+256) and then replaced with a medium containing 100 mM of taurine. After 24 h, the cells were subjected to luciferase assay. The results are expressed as relative values, with a control value of +122/+256 as 1. Each value represents the mean ± S.E. (*n* = 3), * *p* < 0.05, vs. the control value (Student’s *t*-test).

Based on the findings that the taurine response element is contained in the TXNIP promoter region between +143 and +256 (Figure 1), we next constructed a reporter vector containing the promoter region of (+122/+256) and examined the effect of taurine on the promoter activity of TXNIP (+122/+256). A reporter vector containing the promoter region (−1299/+142) was used as a negative control. The promoter activity of TXNIP (+122/+256) was significantly increased by taurine, whereas that of TXNIP (−1299/+142) did not change (Figure 2). This result confirmed that the taurine response element exists in the TXNIP promoter (+122/+256).

**Figure 3:**
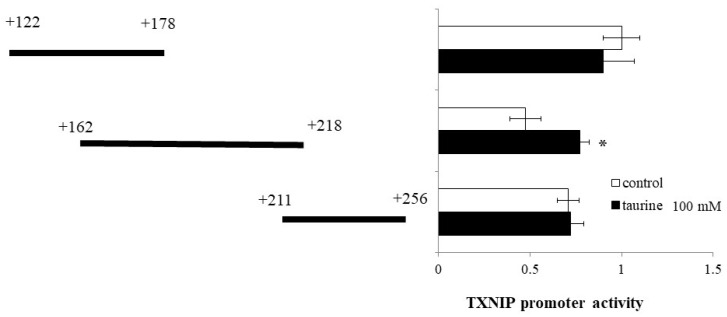
Effect of taurine on the transcriptional activity of the TXNIP promoter between +122/+256. Caco-2 cells were transfected with a reporter vector containing partial promoter regions of TXNIP (+122/+178, +162/+218, +211/+256) and then replaced with a medium containing 100 mM of taurine. After 24 h, the cells were subjected to luciferase assay. The results are expressed as relative values, with a control value of +122/+178 as 1. Each value represents the mean ± S.E. (*n* = 3), * *p* < 0.05, vs. the control value (Student’s *t*-test).

Then, the TXNIP promoter region (+122/+256) was divided into three regions (+122/+178, +162/+218, and +211/+256) and luciferase vectors each containing one region were constructed. Taurine significantly increased the promoter activity of TXNIP (+162/+218) (Figure 3), suggesting that the taurine response element is contained in the TXNIP promoter region (+162/+218).

**Figure 4:**
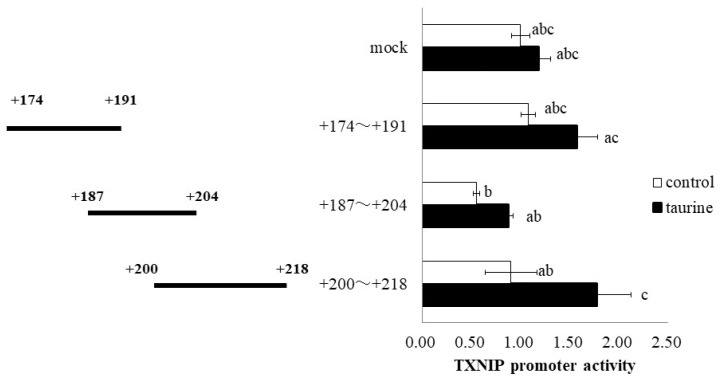
Effect of taurine on the transcriptional activity of the TXNIP promoter between +174/+218. Caco-2 cells were transfected with a reporter vector containing partial promoter regions of TXNIP (+174/+191, +187/+204, +200/+218) and then replaced with a medium containing 100 mM of taurine. After 24 h, the cells were subjected to a luciferase assay. The results are expressed as relative values, with the control value as 1. Each value is the mean ± S.E. (*n* = 3) and the ^abc^ values indicated by different characters are significantly different from each other, and the ^abc^ values indicated by the same characters are not significantly different (Tukey’s test; *p* < 0.05).

**Figure 5:**
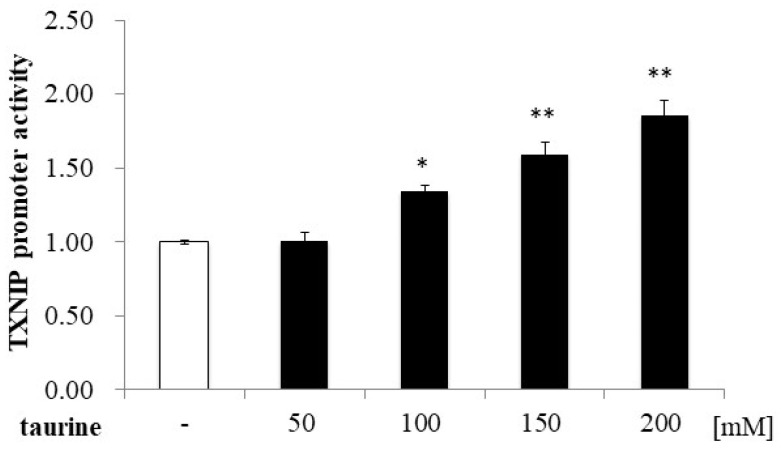
Dose-dependence of taurine-induced TXNIP promoter activity (+200/+218). Caco-2 cells were transfected with a reporter vector containing the partial promoter region of TXNIP (+200/+218) and then replaced with a medium containing 0–200 mM of taurine. After 24 h, the cells were subjected to luciferase assay. The results are expressed as relative values, with a control value of 1. Each value represents the mean ± S.E. (*n* = 3), * *p* < 0.05, ** *p* < 0.01, vs. the control value (Dunnett’s test).

Further, the TXNIP promoter region (+162/+218) was divided into three regions (+174/+191, +187/+204, and +200/+218), and a luciferase vector containing each region was constructed. Taurine significantly increased the promoter activity of TXNIP (+200/+218) (Figure 4), suggesting that the taurine response element is contained in the TXNIP promoter region (+200/+218). Taurine also increased the promoter activity of TXNIP (+200/+218) in a dose-dependent manner (Figure 5), confirming that a taurine response element exists in the promoter region (+200/+218).

**Figure 6:**
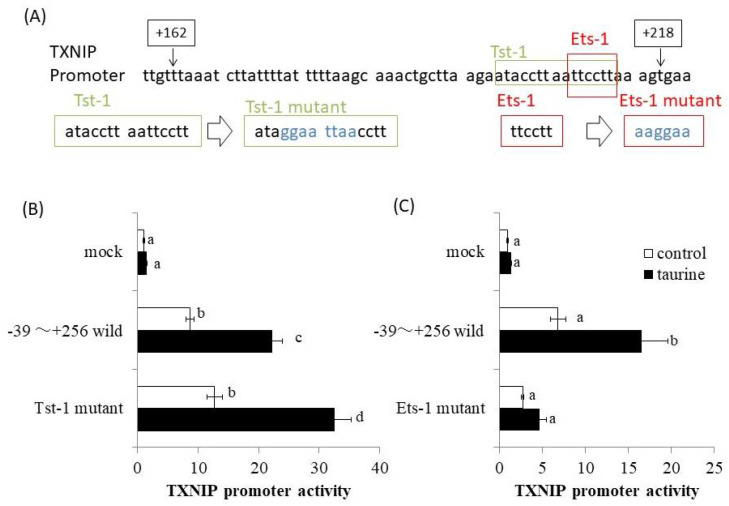
TXNIP promoter sequence (+162/+218) and the effect of taurine on TXNIP promoter activity by employing site-directed mutagenesis. (**A**) Human TXNIP promoter sequences between +162 and +218 are shown. The sequence of putative Tst-1 and Ets-1 response sequences and their mutants are shown, respectively. (**B**,**C**) Caco-2 cells were transfected with a reporter vector containing the partial promoter region of TXNIP (−39/+256; wild type, Tst-1 mutant, Ets-1 mutant) and then replaced with the medium containing 100 mM of taurine. After 24 h, the cells were subjected to the luciferase assay. Results are expressed as relative values with the control value of mock as 1. Each value is the mean ± S.E. (*n* = 4) and the ^abcd^ values indicated by different characters are significantly different from each other and the ^abcd^ values indicated by the same characters are not significantly different (Tukey’s test; *p* < 0.05).

The promoter region of human TXNIP (+162/+218) was analyzed using a bioinformatics approach involving the TRANSFAC database. At the same time, deletion analysis of human TXNIP promoter was also performed. Then, the result of Figure 4 was obtained and the taurine-response region was narrowed down to +200/+218. Therefore, we especially analyzed the TXNIP promoter region (+200/+218) using TRANSFAC database and focused on Tst-1 and Ets-1 as candidates involved in taurine-induced transcription. We then constructed the human TXNIP (+211/+256) promoter vector with site-directed mutagenesis of putative Tst-1 and Ets-1 response elements, respectively (Figure 6A). The results showed that the mutation of the Tst-1 response element had no effect on the increase in TXNIP promoter activity induced by taurine (Figure 6B), but the mutation of the Ets-1 response element abolished the induction of TXNIP promoter activity by taurine (Figure 6C). This result strongly suggests that Ets-1 is involved in taurine-induced upregulation of TXNIP promoter activity.

### 2.2. Effect of Taurine on Ets-1 Binding to TXNIP Promoter (ChIP Assay)

**Figure 7:**
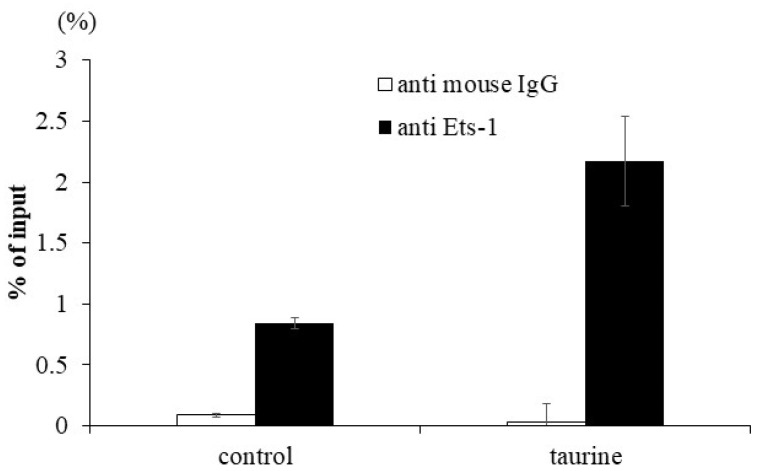
Effect of taurine on the binding of Ets-1 to the TXNIP promoter. Caco-2 cells were cultured in medium containing 100 mM of taurine. After 48 h, a ChIP assay was performed as described in Materials and Methods. The results are expressed as relative values with 100% being the DNA value before immunoprecipitation. Each value represents the mean ± S.E. (*n* = 2).

To confirm the enhancement of Ets-1 binding to the human TXNIP promoter region by taurine, a ChIP assay was performed. Caco-2 cells were cultured with 100 mM of taurine for 48 h and the cells were recovered. The lysate was immunoprecipitated using an anti-Ets-1 antibody. A real-time polymerase chain reaction (PCR) analysis was performed to determine whether the precipitated DNA contained the TXNIP promoter region with the Ets-1 response element. The results showed that taurine clearly increased Ets-1 binding to the TXNIP promoter region (Figure 7). We did not perform experimental verification of Tst-1 binding but only focused our attention on Ets-1.

### 2.3. Effect of Taurine on Ets-1 Activation (Phosphorylation)

**Figure 8:**
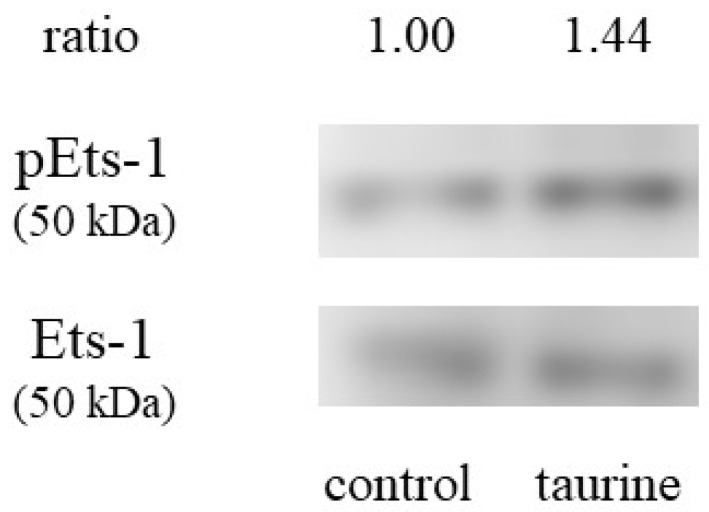
Effect of taurine on the phosphorylation (activation) of Ets-1. Caco-2 cells were cultured in a medium containing 100 mM of taurine for 3 h. The cell lysate was recovered and used for the western blot analysis. The bands were quantified using an image gauge.

We also examined whether taurine activates Ets-1 or not. Ets-1 is activated by Thr38 [23]. Caco-2 cells were incubated with 100 mM of taurine for 3 h and the cell lysate was recovered and used for western blot analysis. Figure 8 shows that taurine increased the protein expression of phosphorylated Ets-1, suggesting that taurine activates Ets-1.

### 2.4. The Involvement of MAP Kinase Family on Taurine-Induced Induction of TXNIP mRNA

**Figure 9:**
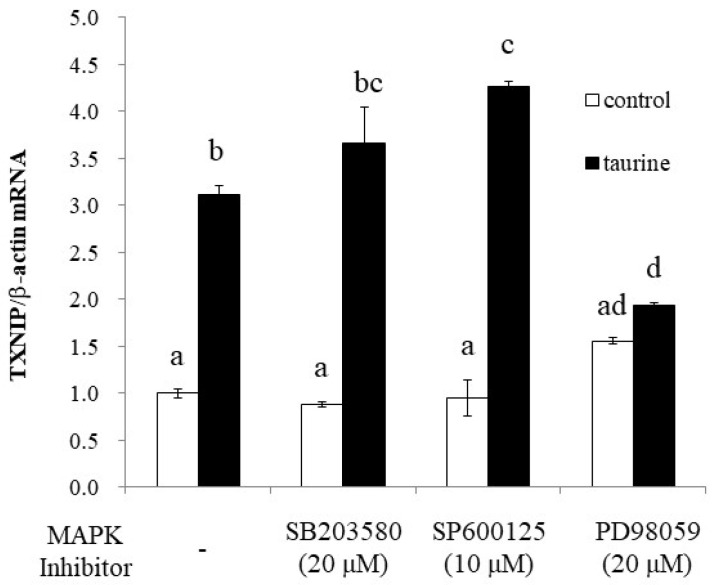
Effect of MAP kinase inhibitors on the taurine-induced increase in TXNIP mRNA in Caco-2 cells. Caco-2 cells were pretreated with each of the three MAPK inhibitors for 2 h and then cultured in medium containing 100 mM of taurine and each inhibitor. After 48 h, RNA was extracted and used for real-time PCR. The results are expressed as relative values, with the control value without the inhibitor as 1. Each value is the mean ± S.E. (*n* = 3) and the ^abcd^ values indicated by different characters are significantly different from each other, and the ^abcd^ values indicated by the same characters are not significantly different (Tukey’s test; *p* < 0.05).

To further elucidate the signaling pathway involved in taurine-induced TXNIP induction, we examined the effect of MAPK inhibitors on taurine TXNIP mRNA expression. The results showed that PD98059, an ERK1/2 pathway inhibitor, significantly suppressed the taurine-induced increase in TXNIP mRNA, whereas the p38 (SB203580) and JNK (SP600125) inhibitors did not (Figure 9).

### 2.5. Effect of Taurine on ERK1/2 Activation in Caco-2 Cells

**Figure 10:**
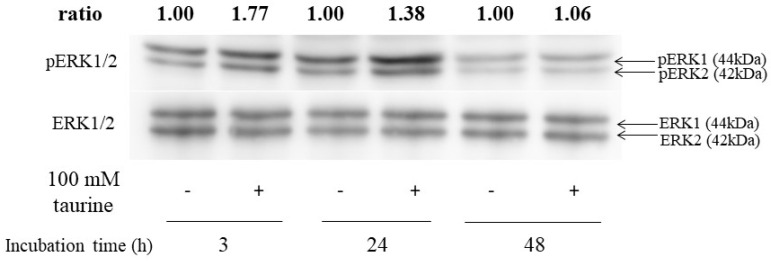
Effect of taurine on the phosphorylation (activation) of ERK1/2. Caco-2 cells were cultured in medium containing 100 mM of taurine for 3, 24, and 48 h. The cell lysate was recovered and used for the western blot analysis. The bands were quantified using an image gauge. Changes in expression are presented as relative values, with the expression level in the control at each treatment time as 1.

Next, to assess whether ERK1/2 was activated by taurine, the western blot analysis was performed. ERK1/2 was phosphorylated after 3 or 24 h of incubation with 100 mM of taurine (Figure 10). These results indicated that taurine activated ERK1/2 in Caco-2 cells.

### 2.6. Involvement of ERK Signaling Pathway in Taurine-Induced Enhancement of Transcriptional Activity of TXNIP

**Figure 11:**
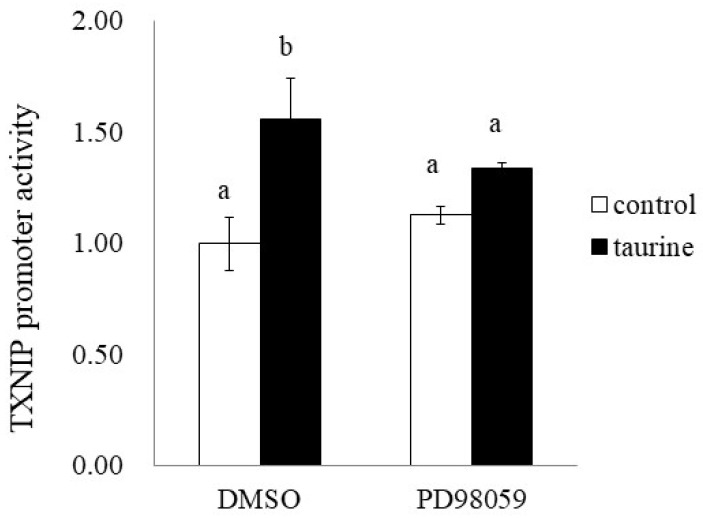
Effect of ERK1/2 cascade inhibitor on the taurine-induced increase in TXNIP promoter. After transfection with a reporter vector containing the promoter region of TXNIP (+200/+218), Caco-2 cells were pretreated with 50 µM PD98059 for 2 h and then cultured in medium containing 100 mM of taurine and the inhibitor. After 24 h, the cells were subjected to a luciferase assay. The results are expressed as relative values, with the control value without inhibitor as 1. Each value is the mean ± S.E. (*n* = 3) and the ^ab^ values indicated by different characters are significantly different from each other and the ^ab^ values indicated by the same characters are not significantly different (Tukey’s test; *p* < 0.05).

Finally, we examined the effect of PD98059 on the taurine-induced increase in TXNIP promoter activity (+200/+218). PD98059 significantly suppressed the taurine-induced increase in TXNIP promoter activity (Figure 11). This result suggests that taurine activates the TXNIP promoter activity via the ERK cascade.

## 3. Discussion

In the present study, we performed a detailed analysis of the mechanisms underlying the taurine-induced enhancement of TXNIP transcriptional activity. Our results demonstrate that taurine activates (phosphorylates) the transcription factor Ets-1 via the ERK signaling pathway and that the activated Ets-1 enhances the transcriptional activity of TXNIP by binding to the response sequence at +200/+218 in the TXNIP promoter region.

A total of 100 mM of taurine increases the TXNIP promoter activity (+200/+218) about two-fold in Figure 4, whereas taurine increases the promoter activity (+200/+218) about 1.3 fold in Figure 5. The difference in the induction ratio of luciferase activity by taurine between Figure 4 and Figure 5 is thought to be due to the difference in the condition of Caco-2 cells. Caco-2 cells are used as an intestinal epithelial model, but this cell line is heterogeneous and it is well known that the cell characteristics and cell response are often changed during passage [24,25]. Although the induction ratio is different, the significant increase of TXNIP promoter activity (+200/+218) by taurine is observed in both Figure 4 and Figure 5.

The deletion analysis of the TXNIP promoter suggests that the response sequences of the transcription factors Tst-1 and Ets-1 in the 200/+218 region of the TXNIP promoter were the candidates for taurine response sequences. However, there have been no reports of promoter analyses on TXNIP in region 3′ from −39, and it is unknown what kind of transcription factor response sequences exist in this region. Therefore, we analyzed the sequences using JASPAR, a database of predicted transcription factor-binding sequences. These results revealed the presence of a putative Tst-1 transcription response sequence and an Ets-1 transcription factor response sequence in +210/+215 of the TXNIP promoter.

Tst-1 belongs to a gene family with a Pit-Oct-Unc (POU) domain, and is reported to be expressed in the brain [26], and represses the expression of the P0 gene, a cell surface adhesion molecule [26], and specifically induces gene expression of the nicotinic acetylcholine receptor (nAchR) subunit 3 [27]. However, Tst-1 has only been examined in the brain and not much information is available about its role in other tissues. Ets-1 is a member of the E26 transformation-specific (Ets) family, which contains a helix-turn-helix DNA-binding region and has been reported to bind to the GGAA/T motif of DNA [28]. The Ets family is reported to be a family of transcription factors involved in diverse physiological functions, such as cell proliferation, differentiation, and apoptosis [29], and Ets-1 is known to be expressed in many tissues [30]. Therefore, we constructed luciferase vectors with mutations in the response sequences of each transcription factor (Tst-1 and Ets-1) and proceeded with the analysis.

Mutation analysis suggests the involvement of Ets-1 in taurine-induced enhancement of TXNIP transcription. Subsequently, we examined whether taurine activates Ets-1 and found that taurine addition activated (phosphorylated) Ets-1 (Figure 8). Considering that Ets-1 is reported to be activated by ERK [31], we hypothesized that taurine phosphorylates the transcription factor Ets-1 via ERK and enhances TXNIP transcriptional activity. A ChIP assay was performed on Caco-2 cells cultured in taurine-containing medium as shown in Figure 7; the results showed that taurine enhanced Ets-1 binding to the TXNIP promoter region. Notably, the binding of Ets-1 to the TXNIP promoter region was observed even in controls, suggesting that Ets-1 is involved in TXNIP transcriptional activity, even in the steady-state. This result is consistent with the results of the mutation analysis shown in Figure 6. A previous study reported that Ets-1 binds to the TXNIP promoter region at a steady-state in the regulation of TXNIP expression in pancreatic cells [32]. Further, Ets-1 has been shown to bind to the Flvcr1 promoter region in Caco-2 cells [33].

These findings suggest that taurine activates transcription factor Ets-1 via ERK and binds to the TXNIP promoter region, resulting in increased TXNIP transcriptional activity. The involvement of Ets-1 in the transcriptional activity of TXNIP has previously been reported in studies that observed induction of TXNIP by synthetic retinoids in osteosarcoma cells [34] and in the regulation of TXNIP expression in pancreatic cells [32]. However, considering that these two previous studies have shown that Ets-1 binds to the response sequence in the TXNIP promoter −300/−400 region, and our results that taurine-induced enhancement of TXNIP transcriptional activity occurs in the TXNIP promoter +200/+218 region, it appears that taurine may activate Ets-1 through multiple mechanisms. In addition to TXNIP, biomolecules that Ets-1 induces include connective tissue growth factor (CTGF/CCN2), a factor that promotes bone formation [35,36], vascular endothelial growth factor (VEGF) [37], parathyroid hormone-related peptide (PTHrP) [38], and p16 [39]. In addition, Ets-1 interacts with other transcription factors such as prox1, SP-1, and AP-1 to induce their expression [37,38]. Therefore, we cannot rule out the involvement of factors other than Ets-1 in the enhancement of TXNIP transcriptional activity by taurine.

**Figure A1:**
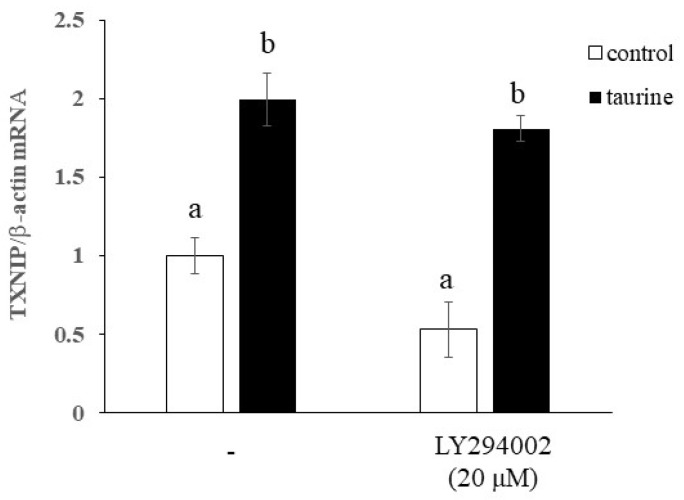
Effect of the PI3K inhibitor on taurine-induced increase in TXNIP mRNA in human hepatic HepG2 cells. HepG2 cells were pretreated with 20 μM LY294002 for 2 h and then cultured in medium containing 100 mM of taurine and the inhibitor. After 48 h, RNA was extracted and used for real-time PCR. The results are expressed as relative values, with the control value without LY294002 as 1. Each value is the mean ± S.E. (*n* = 4), and the ^ab^ values indicated by different characters are significantly different from each other and the ^ab^ values indicated by the same characters are not significantly different (Tukey’s test; *p* < 0.05).

Ets-1 is expressed in all tissues during embryonic development in mice and is reported to be crucial for morphogenesis [40]. In this process, Ets-1 plays a particularly important role in angiogenesis, and it has been reported that double-mutant mice with Ets-2 die in the early stages of angiogenesis [41]. As Ets-1 is involved in the formation of many tissues, it is likely that Ets-1 aids the key function of taurine during fetal, neonatal, and infant life. It has also been reported that natural killer cells do not mature in Ets-1-deficient mice [42]. Thus, Ets-1 appears to be involved in various physiological functions and likely regulates the diverse physiological effects of taurine. Our study elucidated the mechanism of taurine-induced enhancement of TXNIP expression and indicates that this occurs via the ERK pathway. However, the mechanism by which taurine activates ERK is not clear. Upstream signals of the ERK pathway include Ras/Raf/MEK/ERK [43], Ras/PKC/MEK/ERK [44], PKC/Ras/MEK/ERK [45], and PKC/MEK/ERK without Ras and Raf [46]. Since the PKC pathway is involved in three of these four pathways, future studies should examine the effect of the PKC inhibitor Ro318220 on taurine-induced increases in TXNIP mRNA expression. As PKC has also been reported to inhibit TAUT activity [47], it is possible that the cellular uptake of taurine is affected by it, though this possibility needs to be tested. Ras is involved in the activation of Ets-1 [48] and Ets-1 is activated by the Ras/Raf/MEK pathway, particularly in the induction of p16 expression [39]. Therefore, it is likely that taurine activates Ras, leading to the activation of ERK. Consequently, to clarify the upstream factors of the ERK pathway, it is necessary to first examine in detail whether taurine activates PKC and Ras. Further, it is reported that the inhibition of the ERK1/2 pathway by PD98059 in vitro leads to compensatory upregulation of the PI3K/Akt signaling pathway [49]. Therefore, it cannot be ruled out that PI3K/Akt activation is involved in this TXNIP induction. However, we recently found that taurine increases TXNIP mRNA expression in human hepatic HepG2 cells as well as in Caco-2 cells and further revealed that LY294002, a specific PI3K inhibitor, had no significant effect on taurine-induced increase in TXNIP mRNA (Figure A1). Therefore, it is not likely that PI3K/Akt signaling pathway is involved in this regulation.

Caco-2 cells are thought to recognize intracellular or extracellular taurine via unknown taurine receptors or binding proteins, leading to ERK-Ets-1 activation. In the brain, taurine binds to GABA_A_ [50,51,52] and glycine [52,53] receptors and functions as an agonist. However, its binding affinity to the receptors is weaker than that of GABA and glycine. There have been no reports of binding to these receptors in tissues other than cranial nerves. Furthermore, taurine, but neither GABA nor glycine, contributes to the regulation of TAUT expression in intestinal epithelial cells [5]. TAUT receptors are expressed in the plasma membrane [54] and are activated by β-alanine [55]. Noticeably, in the present study, mRNA expression of TXNIP was not induced by β-alanine [9]; therefore, TAUT receptors are unlikely to be involved in the taurine-induced upregulation of TXNIP transcription. Furthermore, the expression of TAUT receptors has been reported only in neural tissues, and the DNA microarray results from our laboratory showed that their expression was absent from our cell cultures [9], suggesting that they are not expressed in Caco-2 cells. These findings suggest that there may be other receptors in cells that specifically recognize taurine; however, these receptors are yet to be identified.

In the present study, we revealed that taurine induced the transcriptional activity of TXNIP via the ERK and Ets-1 signaling pathways. These findings will hopefully contribute to the elucidation of the mechanisms underlying the diverse physiological effects of taurine and the discovery of new functions of taurine.

## 4. Materials and Methods

### 4.1. Materials

The Caco-2 cell line, derived from human colon cancer tissue, was purchased from the American Type Culture Collection (ATCC, Manassas, VA, USA). Dulbecco’s Modified Eagle’s Medium (DMEM) was purchased from Wako Pure Chemicals (Osaka, Japan). Fetal bovine serum (FBS) was purchased from Sigma-Aldrich (St. Louis, MO, USA). Penicillin-streptomycin (10,000 U/mL and 10 mg/mL in 0.9% sodium chloride) and non-essential amino acids (NEAA) were purchased from Gibco (Gaithersburg, MD, USA). The lipofectamine reagent was purchased from Invitrogen (Carlsbad, CA, USA), and the ExScript RT reagent kit and SYBR Premix Ex Taq for real-time PCR were obtained from Takara Bio (Otsu, Japan). All other chemicals used were of reagent grade and commercially available.

### 4.2. Cell Culture

Caco-2 cells were cultured in a medium consisting of DMEM, 10% FBS (*v*/*v*), 1% NEAA (*v*/*v*), 100 U/mL of penicillin, and 100 µg/mL of streptomycin. The cells were incubated at 37 °C in a humidified atmosphere containing 5% CO_2_. The culture medium was replaced every other day. After reaching confluence, the cells were trypsinized with 0.1% trypsin and 0.02% EDTA in PBS and then subcultured. Caco-2 cells used in this study were between passages 35 and 79.

### 4.3. Plasmid Construct

The human TXNIP reporter vector containing the human TXNIP promoter region (−1299/+256) was inserted into the pGL4-basic vector (Promega, Madison, WI, USA) as previously described [10]. The human TXNIP promoter region (−109/+256, −39/+256, −39/+142, −39/+65, −1299/+142, and +122/+256, respectively) was cloned from the pGL4 reporter vector containing the TXNIP promoter (−1299/+256) by PCR. The PCR product was inserted into the pGL4-basic vector or pGL4-Promoter vector by digesting *Kpn*I and *Hind*III, and the restriction enzyme sequence was introduced into the primers. Primers used for each reporter vector are listed in Table A1. The human TXNIP reporter vector containing the human TXNIP promoter region (+122/+178, +162/+218, +211/+256, +174/+191, +187/+204, and +200/+218) was constructed by ligating the pGL4-Promoter vector with each pair of oligonucleotides and annealing as listed in Table A2 and Table A3.

### 4.4. Transfection and Reporter Assay

Caco-2 cells grown in a 24-well plate to 80% confluency were transiently transfected with 1 μg of the reporter vector and 0.05 μg of pRL-CMV using a lipofectamine reagent. Cells treated with or without 100 mM of taurine for 24 h were washed with PBS and lysed with Passive Lysis Buffer (Promega, Madison, WI, USA). Luciferase activity was measured using the dual-luciferase reporter assay (Promega, Madison, WI, USA) and an LB9507 Lumet luminometer (Berthold Technologies, Bad Wildbad, Germany).

### 4.5. Real-Time PCR Analysis

Total RNA was extracted from Caco-2 cells cultured with or without taurine using Isogen (Nippon Gene, Tokyo, Japan), according to the manufacturer’s instructions. Reverse transcription of the RNA was performed using the PrimeScript RT Reagent Kit (TAKARA, Shiga, Japan), and first-strand cDNA was prepared from 0.5 μg of total RNA) and amplified using a SYBR Green Kit (TAKARA). The real-time PCR denaturation temperature was at 95 °C for 15 min, followed by 40 cycles of denaturation at 95 °C, at 60 °C for 15 s, and an extension at 72 °C for 10 s. The primer sequences were as follows: human TXNIP, 5′-ACGCTTCTTCTGGAAGACCA-3′ (forward), and 5′-AAGCTCAAAGCCGAACTTGT-3′ (reverse); β-actin, 5′-CCAGCACAATGAAGATCAAGA-3′ (forward) and 5′- AGAAAGGGTGTAACGCAACTAA-3′ (reverse). Real-time PCR was run on a LightCycler (Roche Applied Sciences, Penzberg, Germany).

### 4.6. Chip Assay

Caco-2 cells were cultured in 6-well plates and incubated with taurine for 48 h. The cells were then homogenized, and the nuclear extract was prepared using the ChIP assay. The ChIP assay was performed using ChIP-IT Express (Active Motif, Carlsbad, CA, USA), according to the manufacturer’s instructions. To quantify the number of DNA fragments containing the TXNIP promoter region bound by Ets-1 protein, real-time PCR was performed. The primer sequences were as follows: human TXNIP promoter, 5′-TCGGATCTTTCTCCAGCAAT-3′ (forward), and 5′-AAATCGAGGAAACCCCTTTG-3′ (reverse).

### 4.7. Western Blot Analysis

Caco-2 cells were cultured in 6-well plates with or without taurine for several hours. The cells were collected with a cell scraper and suspended in lysis buffer (50 mM HEPES (pH 7.5), 150 mM NaCl, 1% NP-40, 0.5% sodium deoxycholate, 1 mM NaVO_3_, 50 mM NaF, 20 mM β-glycerophosphate, 0.1% inhibitor cocktail (Sigma, St. Louis, MO, USA). The cell homogenate was centrifuged at 20,000× *g* for 10 min at 4 °C. The supernatant was used for western blot analysis as described previously [11]. The protein assay was performed using the Bio-Rad Protein Assay Solution (Bio-Rad, Hercules, CA, USA). The primary antibodies used were rabbit anti-human p-Ets-1 (Thr38) (Abcam, Cambridge, UK) and anti-human Ets-1 (Santa Cruz, Dallas, TX, USA). Rabbit anti-human p-ERK1/2 (Thr202/Tyr204) antibody (Santa Cruz, Dallas, TX, USA) and rabbit anti-human ERK1/2 antibody (Santa Cruz, Dallas, TX, USA) were used as the primary antibodies. The secondary antibody was goat anti-rabbit IgG antibody conjugated to horseradish peroxidase (Amersham PLC, Bucks, UK). The bound antibodies were analyzed using an ECL plus western blotting detection system (GE Healthcare, Boston, MA, USA) and a Lumino Image Analyzer (ImageQuant LAS-4000 miniPR; Cytiva, Krefeld, Germany). Original, whole membrane images were provided for the review process and are available from the journal upon request.

### 4.8. Statistical Analysis

Data are expressed as mean ± SE. Statistical comparisons were performed using the Student’s *t*-test, Dunnett’s test, or Tukey’s test.

## Data Availability

The data supporting the findings of this study are available from the corresponding author, H.S., upon reasonable request due to restrictions on privacy.

## Appendix A

**Table A1:** Primers used for constructing each TXNIP promoter vector.

Region		Primer Sequences (5′→3′)
−1299/+256	forward	CCGGTACCCCAACAAAGAATGAAGAGAGAG
	reverse	GCAAGCTTCTCCAAATCGAGGAAACCC
−109/+256	forward	CCGGTACCAGCCAATGGGAGGGATG
	reverse	GCAAGCTTCTCCAAATCGAGGAAACCC
−39/+256	forward	CCGGTACCCGGGCTACTATATAGAGACG
	reverse	GCAAGCTTCTCCAAATCGAGGAAACCC
−39/+142	forward	CCGGTACCCGGGCTACTATATAGAGACG
	reverse	GCAAGCTTCTAGGTTTTCGAAAAGGCGCC
−39/+65	forward	CCGGTACCCGGGCTACTATATAGAGACG
	reverse	GCAAGCTTCCCCAATTGCTGGAGAAAAG
−1299/+142	forward	CCGGTACCCCAACAAAGAATGAAGAGAGAG
	reverse	GCAAGCTTCTAGGTTTTCGAAAAGGCGCC
+122/+256	forward	CCGGTACCGGCGCCTTTTCGAAAACCTAG
	reverse	GCAAGCTTCTCCAAATCGAGGAAACCC

**Table A2:** Partial TXNIP promoter sequence (+122/+256) of oligonucleotide for annealing.

Region		Primer Sequences (5′→3′)
+122/+178	forward	cggcgccttttcgaaaacctagtagttaatattcatttgtttaaatcttattttata
	reverse	agcttataaaataagatttaaacaaatgaatattaactactaggttttcgaaaaggcgccggtac
+162/+218	forward	cttaaatcttattttatttttaagctcaaactgcttaagaataccttaattccttaaaga
	reverse	agcttctttaaggaattaaggtattcttaagcagtttgagcttaaaaataaaataagatttaaggtac
+211/+256	forward	cccttaaagtgaaataattttttgcaaaggggtttcctcgatttggaga
	reverse	agcttctccaaatcgaggaaacccctttgcaaaaaattatttcactttaaggggtac

**Table A3:** Partial TXNIP promoter (+174/+218) sequence of oligonucleotide for annealing.

Region		Primer Sequences (5′→3′)
+174/+191	forward	cttatttttaagctcaaaca
	reverse	agcttgtttgagcttaaaaataaggtac
+187/+204	forward	ccaaactgcttaagaataca
	reverse	agcttgtattcttaagcagtttgggtac
+200/+218	forward	caataccttaattccttaaaa
	reverse	agctttttaaggaattaaggtattggtac

## References

[1] Huxtable R.J. (1992). Physiological actions of taurine. Physiol. Rev..

[2] Schaffer S.W., Azuma J., Takahashi K., Mozaffari M. (2003). Why is taurine cytoprotective?. Adv. Exp. Med. Biol..

[3] Sturman J.A. (1988). Taurine in development. J. Nutr..

[4] Tochitani S. (2019). Functions of Maternally-Derived Taurine in Fetal and Neonatal Brain Development. Adv. Exp. Med. Biol..

[5] Satsu H., Watanabe H., Arai S., Shimizu M. (1997). Characterization and regulation of taurine transport in Caco-2, human intestinal cells. J. Biochem..

[6] Satsu H., Miyamoto Y., Shimizu M. (1999). Hypertonicity stimulates taurine uptake and transporter gene expression in Caco-2 cells. Biochim. Biophys. Acta.

[7] Mochizuki T., Satsu H., Shimizu M. (2002). Tumor necrosis factor alpha stimulates taurine uptake and transporter gene expression in human intestinal Caco-2 cells. FEBS Lett..

[8] Zhao Z., Satsu H., Fujisawa M., Hori M., Ishimoto Y., Totsuka M., Nambu A., Kakuta S., Ozaki H., Shimizu M. (2008). Attenuation by dietary taurine of dextran sulfate sodium-induced colitis in mice and of THP-1-induced damage to intestinal Caco-2 cell monolayers. Amino Acids.

[9] Gondo Y., Satsu H., Ishimoto Y., Iwamoto T., Shimizu M. (2012). Effect of taurine on mRNA expression of thioredoxin interacting protein in Caco-2 cells. Biochem. Biophys. Res. Commun..

[10] Kaimul A.M., Nakamura H., Masutani H., Yodoi J. (2007). Thioredoxin and thioredoxin-binding protein-2 in cancer and metabolic syndrome. Free Radic. Biol. Med..

[11] Kim S.Y., Suh H.W., Chung J.W., Yoon S.R., Choi I. (2007). Diverse functions of VDUP1 in cell proliferation, differentiation, and diseases. Cell Mol. Immunol..

[12] Andres A.M., Ratliff E.P., Sachithanantham S., Hui S.T. (2011). Diminished AMPK signaling response to fasting in thioredoxin-interacting protein knockout mice. FEBS Lett..

[13] Donnelly K.L., Margosian M.R., Sheth S.S., Lusis A.J., Parks E.J. (2004). Increased lipogenesis and fatty acid reesterification contribute to hepatic triacylglycerol stores in hyperlipidemic Txnip-/- mice. J. Nutr..

[14] Hui S.T., Andres A.M., Miller A.K., Spann N.J., Potter D.W., Post N.M., Chen A.Z., Sachithanantham S., Jung D.Y., Kim J.K. (2008). Txnip balances metabolic and growth signaling via PTEN disulfide reduction. Proc. Natl. Acad. Sci. USA.

[15] Oka S., Liu W., Masutani H., Hirata H., Shinkai Y., Yamada S., Yoshida T., Nakamura H., Yodoi J. (2006). Impaired fatty acid utilization in thioredoxin binding protein-2 (TBP-2)-deficient mice: A unique animal model of Reye syndrome. FASEB J..

[16] Sheth S.S., Castellani L.W., Chari S., Wagg C., Thipphavong C.K., Bodnar J.S., Tontonoz P., Attie A.D., Lopaschuk G.D., Lusis A.J. (2005). Thioredoxin-interacting protein deficiency disrupts the fasting-feeding metabolic transition. J. Lipid Res..

[17] van Greevenbroek M.M., Vermeulen V.M., Feskens E.J., Evelo C.T., Kruijshoop M., Hoebee B., van der Kallen C.J., de Bruin T.W. (2007). Genetic variation in thioredoxin interacting protein (TXNIP) is associated with hypertriglyceridaemia and blood pressure in diabetes mellitus. Diabet. Med..

[18] Chutkow W.A., Patwari P., Yoshioka J., Lee R.T. (2008). Thioredoxin-interacting protein (Txnip) is a critical regulator of hepatic glucose production. J. Biol. Chem..

[19] Ferreira N.E., Omae S., Pereira A., Rodrigues M.V., Miyakawa A.A., Campos L.C., Santos P.C., Dallan L.A., Martinez T.L., Santos R.D. (2012). Thioredoxin interacting protein genetic variation is associated with diabetes and hypertension in the Brazilian general population. Atherosclerosis.

[20] Yoshioka J., Schulze P.C., Cupesi M., Sylvan J.D., MacGillivray C., Gannon J., Huang H., Lee R.T. (2004). Thioredoxin-interacting protein controls cardiac hypertrophy through regulation of thioredoxin activity. Circulation.

[21] Takahashi Y., Masuda H., Ishii Y., Nishida Y., Kobayashi M., Asai S. (2007). Decreased expression of thioredoxin interacting protein mRNA in inflamed colonic mucosa in patients with ulcerative colitis. Oncol. Rep..

[22] Satsu H., Gondo Y., Shimanaka H., Watari K., Fukumura M., Shimizu M. (2019). Effect of taurine on cell function via TXNIP induction in Caco-2 cells. Adv. Exp. Med. Biol..

[23] Garrett-Sinha L.A. (2013). Review of Ets1 structure, function, and roles in immunity. Cell. Mol. Life Sci..

[24] Yu H., Cook T.J., Sinko P.J. (1997). Evidence for diminished functional expression of intestinal transporters in Caco-2 cell monolayers at high passages. Pharm. Res..

[25] Steffansen B., Pedersen M.D.L., Laghmoch A.M., Nielsen C.U. (2017). SGLT1-Mediated Transport in Caco-2 Cells Is Highly Dependent on Cell Bank Origin. J. Pharm. Sci..

[26] He X., Gerrero R., Simmons D.M., Park R.E., Lin C.J., Swanson L.W., Rosenfeld M.G. (1991). Tst-1, a member of the POU domain gene family, binds the promoter of the gene encoding the cell surface adhesion molecule P0. Mol. Cell. Biol..

[27] Yang X., McDonough J., Fyodorov D., Morris M., Wang F., Deneris E.S. (1994). Characterization of an acetylcholine receptor alpha 3 gene promoter and its activation by the POU domain factor SCIP/Tst-1. J. Biol. Chem..

[28] Oikawa T., Yamada T. (2003). Molecular biology of the Ets family of transcription factors. Gene.

[29] Sementchenko V.I., Watson D.K. (2000). Ets target genes: Past, present and future. Oncogene.

[30] Dittmer J. (2003). The biology of the Ets1 proto-oncogene. Mol. Cancer.

[31] Roskoski R. (2012). ERK1/2 MAP kinases: Structure, function, and regulation. Pharmacol. Res..

[32] Luo Y., He F., Hu L., Hai L., Huang M., Xu Z., Zhang J., Zhou Z., Liu F., Dai Y.S. (2014). Transcription factor Ets1 regulates expression of thioredoxin-interacting protein and inhibits insulin secretion in pancreatic beta-cells. PLoS ONE.

[33] Fiorito V., Neri F., Pala V., Silengo L., Oliviero S., Altruda F., Tolosano E. (2014). Hypoxia controls Flvcr1 gene expression in Caco2 cells through HIF2alpha and ETS1. Biochim. Biophys. Acta.

[34] Hashiguchi K., Tsuchiya H., Tomita A., Ueda C., Akechi Y., Sakabe T., Kurimasa A., Nozaki M., Yamada T., Tsuchida S. (2010). Involvement of ETS1 in thioredoxin-binding protein 2 transcription induced by a synthetic retinoid CD437 in human osteosarcoma cells. Biochem. Biophys. Res. Commun..

[35] Van Beek J.P., Kennedy L., Rockel J.S., Bernier S.M., Leask A. (2006). The induction of CCN2 by TGFbeta1 involves Ets-1. Arthritis Res. Ther..

[36] Geisinger M.T., Astaiza R., Butler T., Popoff S.N., Planey S.L., Arnott J.A. (2012). Ets-1 is essential for connective tissue growth factor (CTGF/CCN2) induction by TGF-beta1 in osteoblasts. PLoS ONE.

[37] Yoshimatsu Y., Yamazaki T., Mihira H., Itoh T., Suehiro J., Yuki K., Harada K., Morikawa M., Iwata C., Minami T. (2011). Ets family members induce lymphangiogenesis through physical and functional interaction with Prox1. J. Cell Sci..

[38] Itoh T., Ando M., Tsukamasa Y., Akao Y. (2012). Expression of BMP-2 and Ets1 in BMP-2-stimulated mouse pre-osteoblast differentiation is regulated by microRNA-370. FEBS Lett..

[39] Ohtani N., Zebedee Z., Huot T.J., Stinson J.A., Sugimoto M., Ohashi Y., Sharrocks A.D., Peters G., Hara E. (2001). Opposing effects of Ets and Id proteins on p16INK4a expression during cellular senescence. Nature.

[40] Kola I., Brookes S., Green A.R., Garber R., Tymms M., Papas T.S., Seth A. (1993). The Ets1 transcription factor is widely expressed during murine embryo development and is associated with mesodermal cells involved in morphogenetic processes such as organ formation. Proc. Natl. Acad. Sci. USA.

[41] Wei G., Srinivasan R., Cantemir-Stone C.Z., Sharma S.M., Santhanam R., Weinstein M., Muthusamy N., Man A.K., Oshima R.G., Leone G. (2009). Ets1 and Ets2 are required for endothelial cell survival during embryonic angiogenesis. Blood.

[42] Barton K., Muthusamy N., Fischer C., Ting C.N., Walunas T.L., Lanier L.L., Leiden J.M. (1998). The Ets-1 transcription factor is required for the development of natural killer cells in mice. Immunity.

[43] Harrisingh M.C., Lloyd A.C. (2004). Ras/Raf/ERK signalling and NF1. Cell Cycle.

[44] Zhao Y., Liu J., Li L., Liu L., Wu L. (2005). Role of Ras/PKCzeta/MEK/ERK1/2 signaling pathway in angiotensin II-induced vascular smooth muscle cell proliferation. Regul. Pept..

[45] Lee H.Y., Crawley S., Hokari R., Kwon S., Kim Y.S. (2010). Bile acid regulates MUC2 transcription in colon cancer cells via positive EGFR/PKC/Ras/ERK/CREB, PI3K/Akt/IkappaB/NF-kappaB and p38/MSK1/CREB pathways and negative JNK/c-Jun/AP-1 pathway. Int. J. Oncol..

[46] Wen-Sheng W. (2006). Protein kinase C alpha trigger Ras and Raf-independent MEK/ERK activation for TPA-induced growth inhibition of human hepatoma cell HepG2. Cancer Lett..

[47] Loo D.D., Hirsch J.R., Sarkar H.K., Wright E.M. (1996). Regulation of the mouse retinal taurine transporter (TAUT) by protein kinases in Xenopus oocytes. FEBS Lett..

[48] Yang B.S., Hauser C.A., Henkel G., Colman M.S., Van Beveren C., Stacey K.J., Hume D.A., Maki R.A., Ostrowski M.C. (1996). Ras-mediated phosphorylation of a conserved threonine residue enhances the transactivation activities of c-Ets1 and c-Ets2. Mol. Cell. Biol..

[49] Hu C., Huang L., Gest C., Xi X., Janin A., Soria C., Li H., Lu H. (2012). Opposite regulation by PI3K/Akt and MAPK/ERK pathways of tissue factor expression, cell-associated procoagulant activity and invasiveness in MDA-MB-231 cells. J. Hematol. Oncol..

[50] Taber K.H., Lin C.T., Liu J.W., Thalmann R.H., Wu J.Y. (1986). Taurine in hippocampus: Localization and postsynaptic action. Brain Res..

[51] del Olmo N., Bustamante J., del Río R.M., Solís J.M. (2000). Taurine activates GABA(A) but not GABA(B) receptors in rat hippocampal CA1 area. Brain Res..

[52] Hussy N., Deleuze C., Pantaloni A., Desarménien M.G., Moos F. (1997). Agonist action of taurine on glycine receptors in rat supraoptic magnocellular neurones: Possible role in osmoregulation. J. Physiol..

[53] Lewis C.A., Ahmed Z., Faber D.S. (1991). A characterization of glycinergic receptors present in cultured rat medullary neurons. J. Neurophysiol..

[54] Whatley V.J., Harris R.A. (1996). The cytoskeleton and neurotransmitter receptors. Int. Rev. Neurobiol..

[55] Horikoshi T., Asanuma A., Yanagisawa K., Anzai K., Goto S. (1988). Taurine and beta-alanine act on both GABA and glycine receptors in Xenopus oocyte injected with mouse brain messenger RNA. Brain Res..

